# Selective Targeting of Senescent FHs74Int Cells by Human Breast Milk Free Fatty Acids

**DOI:** 10.3390/biology14101355

**Published:** 2025-10-03

**Authors:** Tony Tremblay, Lionel Loubaki

**Affiliations:** 1 Héma-Québec, Medical Affairs and Innovation, Québec, QC G1V 5C3, Canada; tony.tremblay@hema-quebec.qc.ca; 2 Department of Biochemistry, Microbiology and Bioinformatics, Laval University, Québec, QC G1V 0A6, Canada

**Keywords:** human breast milk, senescence, FFA, FHs74, mitochondrial potential

## Abstract

**Simple Summary:**

As babies grow in the womb, their intestines undergo significant changes. Sometimes, specific cells in the intestine stop dividing and enter a state called senescence—a kind of “retirement” for cells. While this can help shape the developing intestine, if these cells aren’t cleared out properly, they might cause problems later in life. To investigate whether human breast milk—already recognized for its support of gut and immune function—could aid in the removal of senescent cells from the developing intestine, lab-grown fetal intestinal cells were exposed to breast milk, infant formula, and various milk components. Cellular responses were analyzed, with particular focus on the impact on senescent cells. Breast milk, unlike formula, appeared to selectively eliminate senescent cells while sparing most non-senescent cells. This effect was attributed to the milk’s fatty fraction, especially free fatty acids (FFAs). When breast milk was warmed, its FFA levels increased, and so did its ability to remove senescent cells. These findings highlight a novel benefit of breast milk: its potential to promote intestinal health by removing aging cells, which may be crucial for long-term gut and immune system function.

**Abstract:**

Cellular senescence is a state of irreversible growth arrest characterized by a pro-inflammatory phenotype, playing dual roles in development. In the fetal intestine, the regulation of senescent cells is critical for maintaining tissue homeostasis. Human breast milk (HBM), known for its rich composition of bioactive molecules, may play a role in modulating senescence, although its effects on senescent intestinal cells remain unexplored. This study investigated whether HBM selectively eliminates senescent cells in the FHs74Int fetal intestinal epithelial cell line. Senescence was assessed via β-galactosidase activity and expression of p16 and p21. The model cell line was treated with HBM, infant formula, and milk fractions, and outcomes included cell recovery, senescence markers, apoptosis, and mitochondrial potential. Total free fatty acids (FFA) were quantified and correlated with senolytic activity. HBM reduced senescent cell recovery without affecting non-senescent cells, correlating with decreased β-galactosidase activity, reduced phospho-p38 and γH2AX expression, mitochondrial depolarization, and caspase activation. Only the lipid fraction retained senolytic activity, which was associated with elevated FFA levels. Incubation of HBM at 37 °C increased FFA content and conferred senolytic activity. These findings are consistent with the idea that HBM exerts selective senolytic effects via FFA, revealing a novel mechanism by which breast milk could contribute to intestinal homeostasis.

## 1. Introduction

Human breast milk (HBM) is a dynamic, bioactive fluid that provides more than just nutrition to the developing infant. Composed of macronutrients (lipids, proteins, and carbohydrates), micronutrients, immunoglobulins, antimicrobial peptides, cytokines, hormones, and extracellular vesicles, HBM is finely tuned to support neonatal development and immune system maturation [1]. Importantly, breast milk is also a source of live cells, including leukocytes and stem-like cells, as well as bioactive microRNAs that can modulate gene expression in recipient intestinal epithelial cells [2]. Studies have demonstrated that breastfed infants have a lower incidence of infectious diseases, allergies, and autoimmune conditions, which has been partly attributed to these immunomodulatory properties [3]. However, the full spectrum of cellular interactions between breast milk and developing fetal tissues, especially in the context of tissue homeostasis and cellular quality control, remains incompletely understood.

Cellular senescence is a state of durable cell cycle arrest triggered by various stressors, including DNA damage, oxidative stress, oncogene activation, and telomere shortening [4,5]. While initially characterized in the context of aging and tumor suppression, senescence is increasingly recognized as playing context-dependent roles during embryonic development [4,5]. During fetal tissue morphogenesis, programmed senescence can help sculpt developing organs by triggering tissue remodeling and paracrine signaling [6]. Although transient senescence is physiologically beneficial during embryogenesis, the persistence of senescent cells can be deleterious, contributing to chronic inflammation, tissue dysfunction, and impaired regeneration [7]. Recent studies suggest that senescent cells can arise in the fetal intestine in response to in utero stressors, such as hypoxia, inflammation, or microbial dysbiosis [8,9]. The accumulation of such cells in the immature gut may compromise epithelial integrity, barrier function, and immune regulation—factors that are critical for preventing intestinal pathology in neonates.

The developing intestine is particularly sensitive to environmental signals that modulate cellular turnover, immune balance, and microbial interactions. Given the complex composition of HBM, it is plausible that breast milk plays a role in regulating the abundance and function of senescent cells in the neonatal gut. Although direct evidence has been lacking recently, several components of HBM have been individually implicated in modulating cellular stress responses. For example, free fatty acid (FFA) and α-lactalbumin-oleic acid complexes in preterm human milk can induce apoptosis in epithelial cells [10]. Additionally, lactoferrin has been shown to modulate DNA damage responses and cell survival pathways in epithelial cells [11]. These findings suggest that HBM may contain natural factors capable of selectively clearing senescent cells, thereby contributing to tissue homeostasis.

This study explores the hypothesis that HBM contains factors capable of selectively eliminating senescent intestinal cells, thereby contributing to neonatal intestinal tissue homeostasis. Experimental findings demonstrate that HBM exerts a selective senolytic effect on senescent fetal intestinal epithelial cells. Exposure to HBM significantly reduced the viability and β-galactosidase activity of senescent cells, while preserving the viability of non-senescent counterparts. This selective effect was associated with mitochondrial depolarization, reduced caspase activation, and decreased DNA damage in the remaining cell population. Notably, the lipid fraction of HBM—particularly its FFA content—was identified as the principal contributor to this selective cytotoxicity.

## 2. Materials and Methods

### 2.1. Human Donor Milk Collection

This study has been approved by Héma-Quebec’s research ethics committee (CER2017-002), and all participants in this study have signed an informed consent. Human donor milk samples were provided by the Héma-Québec breast milk bank as well as by the regulatory analysis department of Héma-Québec. The samples consisted of individual pasteurized and unpasteurized donor milk aliquots that had been stored at −20 °C. The average storage time was approximately two years. Pasteurization was performed using the Holder method (62.5 °C for 30 minutes (min)), which is the standard technique employed by our milk bank to ensure microbial safety.

### 2.2. FHs74 Intestinal Cell Culture

The FHs74 Int cells (FHs74) were obtained from the American Type Culture Collection (ATCC; CCL-241; Manassas, VA, USA). These cells were extracted from the small intestine and thus are composed of a mixed population of epithelial cells, including enterocytes, paneth cells, and potentially some stem cells [12,13,14]. FHs74 Int cells were cultured in Iscove’s Modified Dulbecco’s Medium (IMDM) (Thermo Fisher Scientific, Waltham, MA, USA), at 37 °C, 5% CO_2_ supplemented with 20% qualified fetal bovine serum (FBS; Thermo Fisher), GlutaMAX 1X (Thermo Fischer), 10 μg/mL insulin (from bovine pancreas; Sigma Life Science, Oakville, ON, Canada) and 30 ng/mL of recombinant human epidermal growth factor (EGF, Thermo Fisher) to generate a master cell bank. Briefly, cells were seeded at a density of 6000 cells/cm^2^ in T175 flasks for cell culture. The medium was changed after four days. At day seven of culture, cells were washed with Dulbecco’s Phosphate Buffered Saline (DPBS, Thermo Fisher) and harvested using TrypLE Express (Thermo Fisher) for 20 min at 37 °C, 5% CO_2_. The cell suspension was diluted by adding an equal volume of Plasma-Lyte A (Baxter Canada, Mississauga, ON, Canada) containing 10% Human Serum Albumin (HSA) (Alburex^®^ 25, CSL Behring, Ottawa, ON, Canada). Cells were centrifuged (500× *g*, 10 min), suspended in Plasma-Lyte A containing 5% HSA, and the cell suspension was analyzed (count and viability) on Nucleocounter^®^ NC-250TM (ChemoMetec, Lincoln, NE, USA). FHs74 cells were then reseeded as described above for one or two additional passages and kept frozen in liquid nitrogen vapor phase until use. In all experiments, FHs74 were used at P4.

### 2.3. Assessment of Senescence

The presence of senescence in cultured cells was assessed using Cayman’s Senescence-Associated β-Galactosidase staining kit according to the manufacturer’s instructions (Cayman Chemical Company, Ann Arbor, MI, USA). Flow cytometry analysis of p16 INK4a and p21 was performed using unconjugated anti-p16 INK4a and unconjugated p21 Waf1/Cip1 (F-5), both from Santa Cruz Biotechnology (Dallas, TX, USA). FITC-conjugated anti-mouse IgG (Thermo Fisher) was used as a secondary antibody, and the data were acquired using the BD Accuri C6 (BD Biosciences, San Jose, CA, USA). Furthermore, Pyronin Y/7-AAD (Thermo Fisher) dual staining was performed to determine the cell cycle phase of FHs74, by flow cytometry using the BD Accuri C6 (BD Bioscience). In addition to this latter, we also used the beta-glo assay system to quantify β-Galactosidase expression in mammalian cells (Promega, Madison, WI, USA). Briefly, FHs74 Int cells were seeded in a 96-well plate (Greiner Bio-One; Monroe, NC, USA) at a density of 31,250 cells/cm^2^ for at least 24 h in complete culture medium. For each well, the medium was removed and 100 µL of DPBS was added before the addition of 100 µL of beta-glo reagent. The plate was agitated for 60 s on a plate shaker at 500 rpm and then incubated for 30 min at room temperature (RT) protected from light. The luminescence signal was then measured using the Synergy H1 multimode reader (Biotek; Agilent, Santa Clara, CA, USA). Results of this quantification are presented as a percentage of the signal, with the untreated condition serving as the maximum β-glo signal under our experimental conditions.

### 2.4. FHs74 Int Cells Exposure to Human Breast Milk

All assays were performed using FHs74 cells at passage four. Thus, upon thawing using Thawstar (BIOLIFE Solutions; Bothell, WA, USA) and centrifugation (500× *g*, 10 min), cells were counted and suspended in a complete IMDM culture medium. 2 × 10^5^ cells per well were seeded onto a 24-well plate and allowed to adhere for at least 18 h at 37 °C and 5% CO_2_.

The following day, the culture medium was removed and replaced with either HBM or formula milk (FM; Enfamil A+ Enfacare, Mead Johnson Nutrition, Kanata, ON, Canada), both diluted 1:50 in IMDM (Thermo Fisher), and incubated for 2 h at RT with FHs74 cells under the following conditions: FHs74 alone (baseline), FHs74 incubated with formula milk (negative control), and FHs74 incubated with the HBM samples to be tested. Formula milk, though containing bioactive compounds, did not induce senescence or cytotoxicity in our assays, making it a suitable functional negative control. Following incubation, FHs74 cells were washed with DPBS before being harvested and counted using the NC-250 automated cell counter, or for quantification of β-galactosidase activity.

### 2.5. Flow Cytometry

Flow cytometry analysis was used to assess the expression level of both phosphorylated p38 and NFκB. Briefly, cells were washed with DPBS + 1.25 mg/mL human serum albumin (HAS; CLS Behring, Ottawa, ON, Canada) + 0.03% Proclin (Sigma Aldrich, Saint Louis, MO, USA) and then fixed with formaldehyde (Thermo Fisher; 1.5%) for 20 min at RT. After the fixation step, the cells were washed again and then incubated for 20 min in 90% (*v*/*v*) methanol in DPBS on ice to permeabilize them. An additional wash was performed, and cells were labelled, for 20 min, with 20 μL/test of Alexa Fluor 647 (AF647)-conjugated anti-human phospho-p38 (clone pT180/pY182; BD Biosciences) or 5 µL/test of eFluor660-conjugated anti-human phospho-NFκB p65 (clone B33B4WP; Thermo Fisher). After a final wash, cells were suspended in 200 μL of DPBS + 2% FBS before being analyzed using the flow cytometer BD Accuri C6 (BD Bioscience). In addition to p38 and NFκB, we also assessed the expression of caspase 3/7 (Cell eventTM Caspase 3/7 Green flow cytometry assay kit; Thermo Fisher), AF488-conjugated anti-human H2AX (Clone pS139; BD), and the mitochondrial membrane potential (Mitoprobe DiOC_2_(3) 488nm; Thermo Fisher) of FHs74 before and after exposure to HBM.

### 2.6. Quantification of Free Fatty Acids

Total free fatty acid (FFA) concentrations in HBM samples were quantified using the Free Fatty Acid Assay Kit—Quantification, a fluorometric assay according to manufacturer’s instructions (Abcam, Toronto, ON, Canada). The fluorescence was acquired using the Synergy H1 multimode reader (Biotek).

### 2.7. Identification of Human Breast Milk Active Component

HBM samples were separated into aqueous and lipid fractions and analyzed by size exclusion chromatography (SEC). Briefly, milk samples were centrifuged (10,000× *g*, 10 min, 20 °C), and the aqueous fraction was recovered. The same volume of PBS was then added to the lipid fraction for its recovery. Whole HBM, aqueous and lipid fractions were diluted with PBS (1:25) and 0.22 µm filtered. 100 μL was injected into a SEC column (Superose 6 Increase 10/300, Cytiva no. 29091596) at the flow rate of 0.6 mL/min with an ÄKTA purifier 100 (absorbance signal at 214 nm was used). The column had previously been equilibrated with PBS.

In addition, some selected Non-cytotoxic-HBM (Noncyt-HBM) samples were incubated at 37 °C for four days to increase their FFA levels [15,16,17]. After this incubation period, the quantity of FFA was measured and their senolytic capacity evaluated through the β-galactosidase activity measured using the beta-glo assay as described in the previous sections.

### 2.8. Statistical Analysis

All analyses were performed using GraphPad Prism 10 (GraphPad, San Diego, CA, USA). All values are reported as means ± standard errors of the mean, and statistical comparisons were carried out using a paired t-test, a Kruskal–Wallis test (i.e., a nonparametric ANOVA), a Wilcoxon matched-pairs signed-rank test, or a Mann–Whitney test (where applicable). Spearman correlation analysis was performed between fatty acid levels and β-galactosidase signal. A *p*-value below 0.05 was considered statistically significant.

## 3. Results

### 3.1. Human Breast Milk Selectively Eliminates Senescent Cells

**Figure 1:**
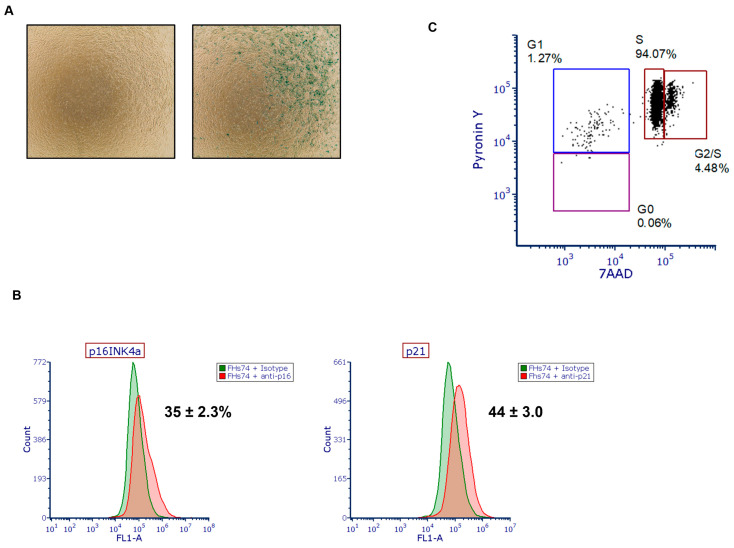
Detection of senescent cells in FHs74 Int cells. (**A**) Representative β-galactosidase staining of FHs74 cells showing the presence of senescent cells. (**B**) Representative histogram of the expression of p16INK4a (left) and p21 (right) assessed by flow cytometry. Data are presented as Mean ± standard error of the mean (SEM), *n* = 4. (**C**) Representative flow cytometry dot plot image of Pyronin Y/7-AAD dual staining of freshly thawed FHs74.

To determine the presence of senescent cells within our population of fetal small intestinal epithelial cells (FHs74), we performed staining using the Senescence β-Galactosidase Staining Kit to detect SA-β-gal activity, along with immunostaining for p16INK4a and p21, two well-established markers of cellular senescence [18,19,20]. The results revealed a substantial proportion of cells positive for these markers, indicating the presence of a senescent subpopulation (Figure 1A,B). Notably and as expected, the levels of p16 and p21 expression were consistent with the intensity of β-galactosidase activity, supporting a strong association between these markers and the actual senescent phenotype in our cell model. In addition, cell cycle phase analysis using Pyronin Y/7-AAD staining revealed that only a small fraction of cells were in G0 (quiescent phase) and G1, while most were in S phase (Figure 1C).

**Figure 2:**
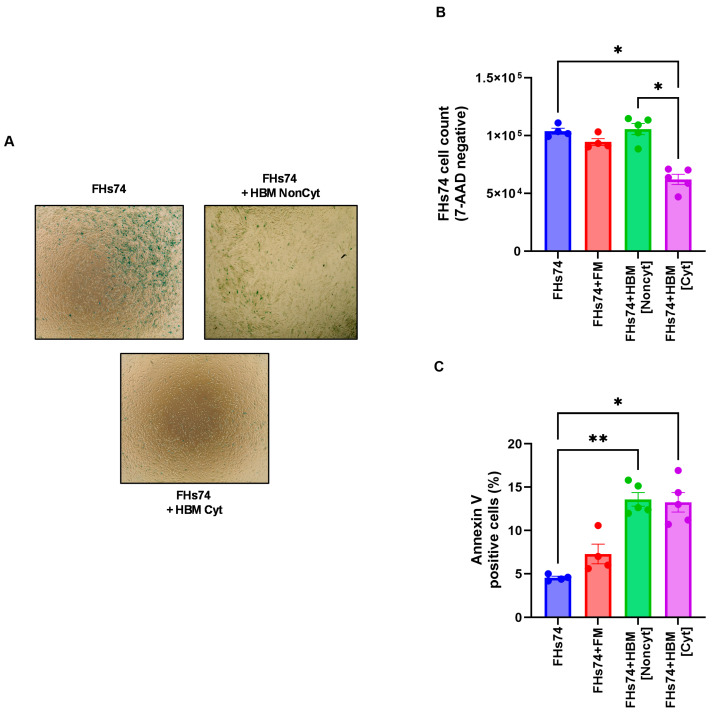
Impact of HBM on FHs74 Int cells. (**A**) Representative β-galactosidase staining of FHs74. cells exposed or not to NonCyt or Cyt-HBM. (**B**) Flow cytometry cell counts of viable (7-AAD negative) FHs74 cells recovered after exposure to different HBM samples. (**C**) Flow cytometry expression of Annexin V. Data are presented as Mean ± standard error of the mean (SEM); * *p* ≤ 0.05, ** *p* ≤ 0.01. *n* = 4–5 different HBM samples. *Note: The difference in sample size between the FHs74-only condition (n = 4) and the HBM-treated conditions (n = 5) is due to the experimental design, in which multiple HBM donors were tested simultaneously against a single FHs74Int control within the same experiment.* FHs74 = FHs74 intestinal cells alone; HBM = Human breast milk; FHs74 + FM = FHs74 intestinal cells exposed to formula milk; FHs74 + HBM = FHs74 intestinal cells exposed to human breast milk.

Exposure of FHs74 cells to HBM showed variable effects on the number of cells recovered post-treatment (Figure 2A,B). Indeed, the analysis of this result allowed us to distinguish two categories of HBM: non-cytotoxic HBM (Noncyt), which did not induce significant cell loss, and cytotoxic HBM (Cyt), which caused a marked decrease in the number of viable FHs74 recovered after exposure (Figure 2B). In addition, we assessed annexin V expression to evaluate membrane destabilization and early apoptotic events. Interestingly, both types of HBM significantly increased annexin V staining (Figure 2C), suggesting that HBM exposure affects membrane integrity regardless of its cytotoxic classification. However, only Cyt-HBM led to substantial cell loss, indicating that while both HBM types may initiate membrane perturbation, only Cyt-HBM drives cell death, likely through a selective mechanism targeting senescent cells.

**Figure 3:**
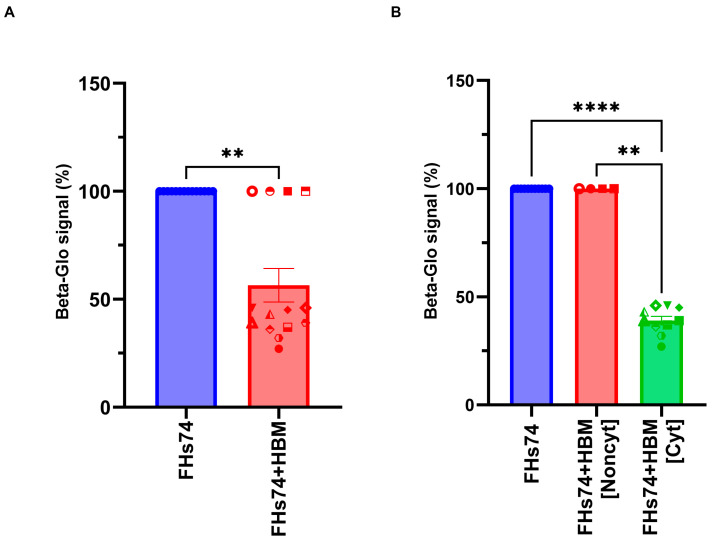
Quantification of β-galactosidase activity in FHs74 cells. (**A**) Luminescence signal measured by the beta-glo assay following exposure to different HBM samples. (**B**) Two categories of HBM based on their β-galactosidase-reducing activity. Data are presented as Mean and standard error of the mean (SEM); ** *p* ≤ 0.01; **** *p* ≤ 0.0001. *n* = 14 different donors of HBM. *Note: Quantification of β-galactosidase activity in FHs74Int cells exposed to different HBM samples. Error bars are not visible for the FHs74Int and Noncyt-HBM conditions due to the absence of variation between samples (100% signal in both cases).* FHs74 = FHs74 intestinal cells alone; HBM = Human breast milk; FHs74 + HBM = FHs74 intestinal cells exposed to human breast milk.

In addition, to further confirm the impact of HBM on FHs74 cells, the beta-glo assay system, a homogeneous method used to quantify β-galactosidase expression in mammalian cells, was employed (Figure 3). The results of this assay closely mirror those obtained from cell viability assays, reinforcing the classification of HBM samples into cytotoxic and non-cytotoxic categories. Cyt-HBM samples consistently showed both a significant reduction in β-galactosidase activity and a marked loss of viable cells, supporting their senolytic potential. In contrast, Noncyt-HBM samples had minimal impact on both cell viability and senescence marker expression.

### 3.2. p38 Expression Level Is Reduced in Recovered FHs74 Cells

**Figure 4:**
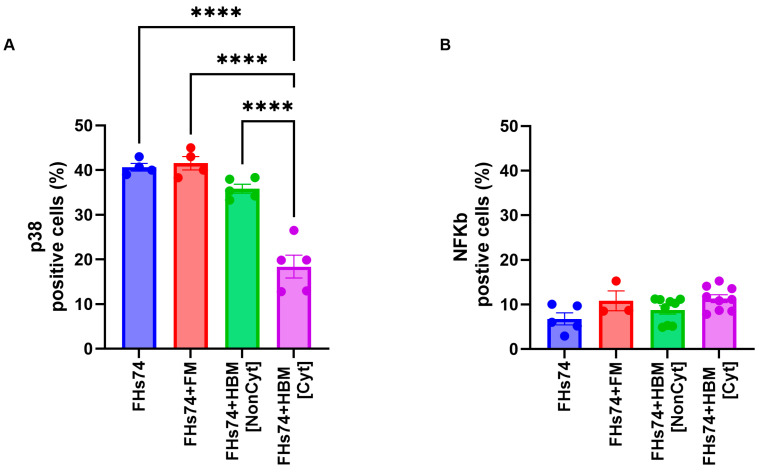
Expression of signaling molecules after HBM exposure. Expression of phosphorylated p38 (**A**) and NFκB (**B**) in FHs74 cells following treatment with Cyt- or Noncyt-HBM. Data are presented as Mean and standard error of the mean (SEM); **** *p* ≤ 0.0001. *n* = 5–9 different donors of human breast milk. *Note: The difference in sample size between the FHs74-only condition (n = 4–5) and the HBM-treated conditions (n = 5–9) is due to the experimental design, in which multiple HBM donors were tested simultaneously against a single FHs74Int control within the same experiment*. FHs74 = FHs74 intestinal cells alone; FHs74 + FM = FHs74 intestinal cells exposed to formula milk; FHs74 + HBM = FHs74 intestinal cells exposed to human breast milk.

p38 Mitogen-Activated Protein Kinase (MAPK) and NFκB play a significant role in cellular senescence [21,22]. Therefore, we assessed the expression level of both p38 and NFκB in FHs74 before and after exposure to both Noncyt-HBM and Cyt-HBM. Our results revealed a significant reduction in p38 expression following exposure to Cyt-HBM, whereas no modulation was observed with Noncyt-HBM (Figure 4A). In contrast, NFκB expression levels were not significantly affected by exposure to either group of HBM (Figure 4B).

### 3.3. Apoptotic and Stress Response Associated Signals Are Reduced in Recovered FHs74 Cells

**Figure 5:**
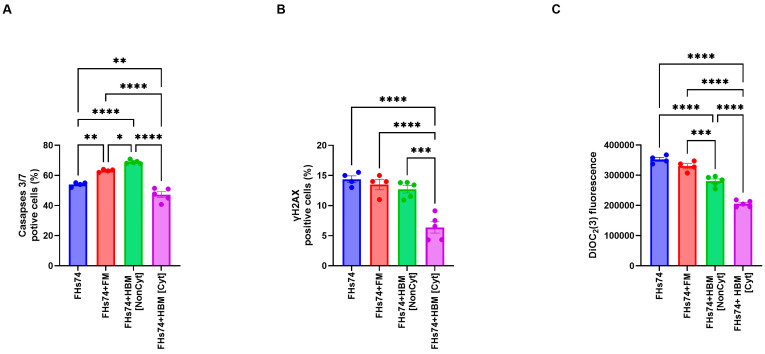
Cellular stress and apoptosis markers. (**A**) Caspase 3/7 activity in FHs74 cells exposed to Cyt- and Noncyt-HBM. (**B**) γH2AX expression levels indicate DNA damage. (**C**) Mitochondrial membrane potential measured by DiOC_2_(3) staining. Data are presented as Mean and standard error of the mean (SEM); * *p* ≤ 0.05; ** *p* ≤ 0.01; *** *p* ≤ 0.001; **** *p* ≤ 0.0001. *n* = 5 different donors of human breast milk. *Note: The difference in sample size between the FHs74-only condition (n = 4) and the HBM-treated conditions (n = 5) is due to the experimental design, in which multiple HBM donors were tested simultaneously against a single FHs74Int control within the same experiment.* FHs74 = FHs74 intestinal cells alone; FHs74 + FM = FHs74 intestinal cells exposed to formula milk; FHs74 + HBM = FHs74 intestinal cells exposed to human breast milk.

Treatment of FHs74 cells with Noncyt-HBM or Cyt-HBM showed different results compared to formula milk. Both Noncyt-HBM had significant effect on caspase activity(Figure 5A). Additionally, the expression of the DNA damage marker γH2AX was reduced considerably in the recovered FHs74 cells following exposure to Cyt-HBM (Figure 5B). Moreover, a significant depolarization of the mitochondrial membrane was observed in these cells, indicating cellular stress (Figure 5C).

### 3.4. The Senolytic Capacity of HBM Is Associated with Its Levels of FFA

**Figure 6:**
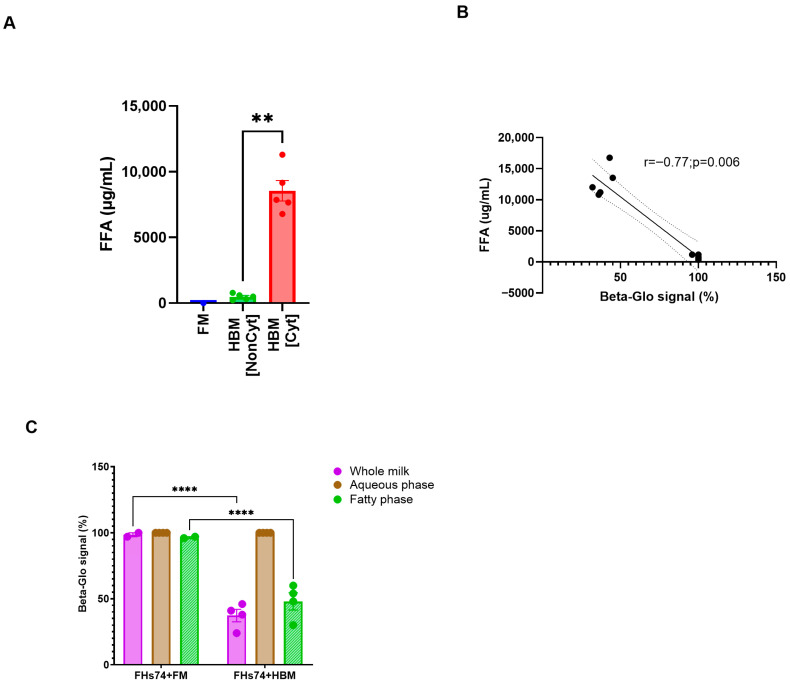
Fatty acid fraction and HBM cytotoxicity. (**A**) Quantification of FFA levels across HBM samples (*n* = 10). (**B**) Correlation between FFA concentration and Beta-glo signal (*n* = 10). (**C**) Beta-glo signal after exposure to whole HBM, aqueous, and fatty fractions (*n* = 4). Data are presented as Mean and standard error of the mean (SEM); ** *p* ≤ 0.01; **** *p* ≤ 0.0001. FHs74 = FHs74 intestinal cells alone; FHs74 + FM = FHs74 intestinal cells exposed to formula milk; FHs74 + HBM = FHs74 intestinal cells exposed to human breast milk.

To determine the potential molecular driver of the senolytic effect observed with specific HBM samples, we first quantified the concentration of FFA in each milk specimen, as it has been reported that FFAs can exert cytotoxic effects on intestinal epithelial cells [10,15,23]. Cyt-HBM samples exhibited significantly higher FFA concentrations compared to Noncyt-HBM and formula milk (Figure 6A). Correlation analysis revealed a strong inverse relationship (r = −0.77; *p* = 0.006) between FFA levels and β-galactosidase signal intensity (Figure 6B), suggesting that elevated FFA concentrations are associated with enhanced elimination of senescent cells. HBM samples containing higher FFA levels consistently showed greater reduction in senescence markers, supporting a role for FFAs as key mediators of the observed senolytic activity.

To confirm this hypothesis, we fractionated selected Cyt-HBM samples into their aqueous and lipid components and evaluated their senolytic potential separately. While neither the aqueous fraction nor any fraction from FM reduced the β-galactosidase activity, the lipid fractions of Cyt-HBM samples retained the full senolytic activity observed with whole milk (Figure 6C). These results confirm that the senolytic effect of HBM is primarily mediated by its lipid fraction, and potentially by its content in FFA.

**Figure 7:**
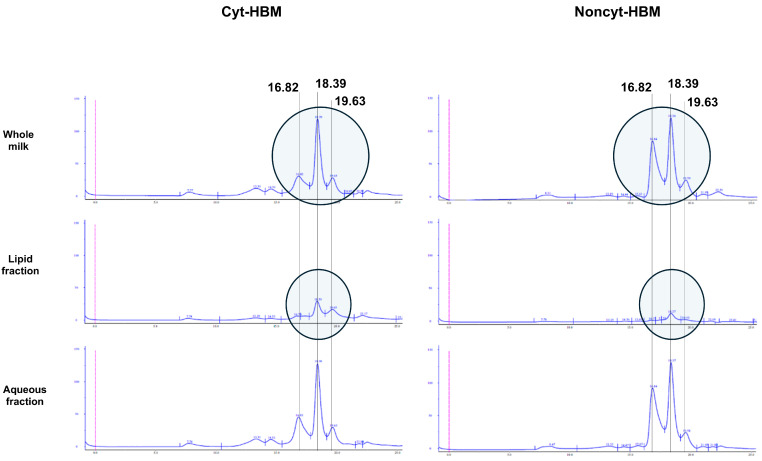
SEC chromatogram of HBM. Representative chromatogram of whole breast milk, aqueous fraction, and fatty fraction from cytotoxic and non-cytotoxic human breast milk.

Since the HBM phases were obtained by centrifugation, SEC analyses were performed to characterize the composition of the various HBM phases. As shown in Figure 7, a reduction in the concentration of a compound eluting at 16.82 mL was observed in the whole Cyt-HBM compared to the entire Noncyt-HBM. The chromatographic profiles of the aqueous fractions also differed between Cyt-HBM and Noncyt-HBM. Finally, in the lipid phase, we noted a marked increase in the compound eluting at 18.39 mL in Cyt-HBM compared to Noncyt-HBM.

**Figure 8:**
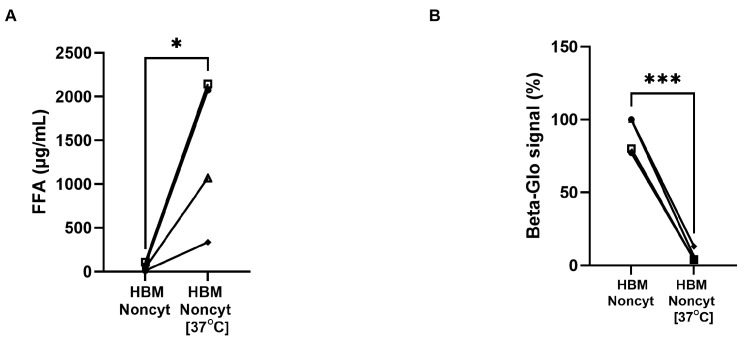
FFA concentration and β-galactosidase signal. (**A**) Quantification of FFA levels in Noncyt-HBM samples prior and after 4 days at 37 °C. (**B**) Beta-glo signal of FHs74 intestinal cells to Noncyt-HBM samples prior and after 4 days at 37 °C. Data are presented as Mean and standard error of the mean (SEM); * *p* ≤ 0.05; *** *p* ≤ 0.001. *n* = 4 different donors of human breast milk. HBM = human breast milk; FFA = Free fatty acids.

Furthermore, it has been reported that HBM contains lipases that may contribute to the generation of FFA within the milk [15,16,17]. To test the hypothesis that FFA is involved in the senolytic effect observed in our study, we incubated selected HBM samples at 37 °C for four days, thereby increasing FFA levels in some initially non-cytotoxic HBMs. Our results showed a substantial increase—approximately 20-fold—in FFA concentration following incubation (Figure 8A). When these incubated Noncyt-HBMs were applied to FHs74 cells, they exhibited senolytic activity, in contrast to their counterparts that had been stored at −20 °C and thawed immediately before use (Figure 8B). These findings support the role of FFA in mediating the senolytic properties of HBM.

## 4. Discussion

In the intestinal tract, the elimination of senescent cells is crucial for maintaining tissue homeostasis and potentially preventing specific pathologies, including necrotizing enterocolitis (NEC). Previous studies have shown that human breast milk (HBM) is essential for neonatal growth and development. Indeed, it contains numerous bioactive factors (growth factors, cytokines, α-lactalbumin, lactoferrin, etc.) that interact with intestinal cells to modulate their fate, including apoptosis [10,15,24]. However, its role in regulating senescent intestinal cells remains unexplored. It may represent a key mechanism in maintaining intestinal homeostasis by promoting the development of the infant’s digestive system. Thus, this study provides evidence that HBM exhibits selective senolytic activity, targeting senescent fetal intestinal epithelial cells.

We first confirmed the presence of senescent cells in FHs74Int cultures through SA-β-galactosidase activity and the expression of p16INK4a and p21, which are well-established markers of senescence [18,19,20]. These findings are consistent with previous observations of senescence in FHs74 cells [25] and align with reports suggesting that senescence occurs naturally in immature tissues during development [26]. This provides a biologically relevant context for studying senescence-targeting mechanisms in the fetal gut.

Exposure to HBM revealed heterogeneity between samples, enabling classification into non-cytotoxic and cytotoxic groups. The criteria for this classification were based on a specific numerical cutoff: Cyt-HBM samples exhibited a reduction in senescent cell recovery and β-galactosidase activity greater than 25%, while non-cytotoxic HBM samples showed reductions below this threshold. This quantitative categorization makes the grouping transparent and supports replication. Only cytotoxic HBM (Cyt-HBM) significantly reduced senescent cell recovery and β-galactosidase activity, while largely sparing non-senescent cells. These findings support a selective senolytic effect, consistent with the emerging concept that certain natural factors can differentially target senescent versus proliferating cells [27,28,29]. Increased Annexin V staining across both groups suggests that HBM perturbs membrane integrity, but only Cyt-HBM induced sufficient stress to eliminate senescent cells.

Mechanistically, Cyt-HBM reduced phosphorylated p38 expression, a key driver of senescence, without affecting NF-κB, suggesting a p38-dependent process [21,27,28,29]. In addition, Cyt-HBM exposure decreased γH2AX and mitochondrial potential, while reducing caspase activity. The absence of strong caspase activation despite significant cell loss raises the possibility of non-apoptotic cell death pathways. We hypothesize that ferroptosis, characterized by iron-dependent lipid peroxidation, could be one such pathway linked to the accumulation of free fatty acids (FFAs) and mitochondrial dysfunction [30]. Our findings of elevated FFA levels in Cyt-HBM, coupled with mitochondrial depolarization, suggest that ferroptosis is a plausible contributor to the observed senolytic effect, warranting further investigation.

A key finding of this study is the correlation between FFA content and the senolytic capacity of HBM. Elevated FFA levels are strongly associated with reduced β-galactosidase activity, and only the lipid fraction reproduced the senolytic effect, in line with prior work reporting cytotoxic roles of FFAs in intestinal epithelial models [15,23,24]. Importantly, lipid fractions from non-cytotoxic HBM were inactive, suggesting that a threshold concentration of FFAs may be necessary to trigger selective elimination of senescent cells. This is further supported by our observation that artificially increasing FFA levels in initially non-cytotoxic HBM samples conferred senolytic activity, strengthening the causal link between FFAs and the observed phenotype.

In addition to FFAs, size exclusion chromatography revealed lipid-associated compounds enriched in Cyt-HBM compared to Noncyt-HBM, suggesting that other lipid-derived molecules may act synergistically with FFAs. Such synergy could explain donor-to-donor variability and aligns with emerging evidence that complex lipid mixtures in breast milk exert multifaceted effects on epithelial and immune function [23,24]. Identifying these compounds will be essential to fully elucidate the mechanisms driving the clearance of senescent cells.

Beyond mechanistic insights, these findings have important biological and clinical implications. Senescent cell accumulation in the fetal intestine has been linked to adverse outcomes, particularly under intrauterine stressors such as hypoxia or inflammation, and may contribute to NEC pathogenesis [8]. By selectively eliminating senescent cells, HBM may support intestinal barrier integrity, epithelial turnover, and immune regulation in neonates, reducing the risk of NEC. The observed inter-donor variability also highlights potential implications for milk bank practices, suggesting that differences in lipid composition could inform donor selection or processing protocols for vulnerable preterm infants.

In summary, our work identifies FFAs as key mediators of the senolytic activity of HBM and suggests the possible involvement of additional lipid-derived compounds. This not only uncovers a novel functional property of HBM but also provides a framework for future studies investigating natural senolytic pathways in neonatal development. Elucidating the mechanisms by which HBM modulates senescent cell clearance could enhance our understanding of neonatal gut health and open avenues for therapeutic strategies targeting senescent cells in both early-life and age-related diseases.

## 5. Conclusions

This study demonstrates that human breast milk (HBM) can selectively eliminate senescent fetal intestinal epithelial cells (FHs74Int) through a mechanism involving FFAs. This senolytic activity is specific, sparing non-senescent cells, and is associated with mitochondrial alterations and reduced DNA damage markers, suggesting a non-apoptotic pathway of cell clearance. The lipid fraction of HBM, particularly in samples with elevated FFA levels, was responsible for this effect, highlighting the importance of milk composition in regulating the neonatal intestine. These findings reveal a previously unrecognized role of HBM in promoting intestinal homeostasis during early development, suggesting that FFAs may act as natural senolytic agents. This opens new avenues for exploring milk-derived lipid components as potential therapeutic tools for managing neonatal intestinal disorders, including NEC, and possibly for broader applications in age-related diseases involving senescent cell accumulation.

## Data Availability

The data presented in this study are available on request from the corresponding author due to legal restrictions.
